# Taking pride in biology

**DOI:** 10.1038/s42003-022-03551-1

**Published:** 2022-06-24

## Abstract

June is LGBTQIA+ Pride Month in the United States, where part of the *Communications Biology* team is based. However, we recognize that Pride Month is just one of many opportunities to celebrate the achievements of this community, and remain committed to using our platform as a journal to amplify and honor queer voices year-round.

For many, Pride Month is a time to celebrate the important contributions made by the LGBTQIA+ community to science and society. However, once Pride ends, LGTBQIA+ researchers will continue their fight for recognition and equality throughout the world, even without the extra stage offered by Pride celebrations. While Pride provides a vital spotlight on the LGBTQIA+ community, queer researchers deserve our support more than 30 days out of the year.

**Figure:**
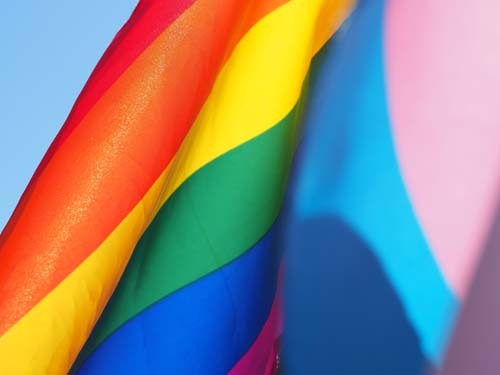
Unsplash

“*Communications Biology* is dedicated to providing a safe platform for queer researchers to share their scientific stories and personal experiences year-round”

As a journal, *Communications Biology* is dedicated to providing a safe platform for queer researchers to share their scientific stories and personal experiences year-round. We are proud to have been able to highlight LGBTQIA+ researchers at multiple career stages, as part of ongoing initiatives like Transgender Day of Visibility (March 31)^1,2^, LGBTQIA+ STEM Day (November 18)^3,4^, and our Q&A series^5^. We encourage our readers to explore these stories in more detail, through our blog series on LGBTQIA+ early-career researchers, as well as our new Q&A Collection highlighting scientists from diverse backgrounds and career stages.

Whenever possible, our editors will also continue to directly engage with the LGBTQIA+ community at larger-scale conferences like the Out in STEM annual meeting, or more local programs like Scientific QUEERies^4^. Even outside of these conferences or symposia, we are committed to provide training opportunities to LGBTQIA+ early-career researchers through peer review, or consult on publications and editorial career options. Moreover, we will continue to advocate and amplify vital programming from organizations like Trans in STEM^5^, Pride in STEM, and 500 Queer Scientists, all of which help promote LGBTQIA+ visibility in the sciences.

The LGBTQIA+ community deserves more than just the visibility provided through Pride, and, while small, we hope that our actions as a journal will help spark discussion (and, importantly, action) on how science as a whole can better support queer researchers.
